# Methodological reflections on the evaluation of the implementation and adoption of national electronic health record systems

**Published:** 2012-06-15

**Authors:** Amir Takian, Dimitra Petrakaki, Tony Cornford, Aziz Sheikh, Nick Barber

**Affiliations:** Brunel University, UK; Sussex University, UK; London School of Economics and Political Sciences, UK; University of Edinburgh, UK; The School of Pharmacy, University of London, UK

**Keywords:** electronic health record, evaluation, methodology, socio-technical changing

## Abstract

**Introduction/purpose of presentation:**

Far-reaching policy commitments to information technology-centered transformations of healthcare systems have now been made in many countries. There is as yet little empirical evidence to justify such decisions, hence the need for rigorous independent evaluation of current implementation efforts. Such evaluations however pose a number of important challenges. This presentation has been designed as a part of a Panel based on our experience of evaluating the National Health Service’s (NHS) implementation of electronic health records (EHR) systems in hospitals throughout England. We discuss the methodological challenges encountered in planning and undertaking an evaluation of a program of this scale and reflect on why and how we adapted our evaluation approach—both conceptually and methodologically—in response to these challenges.

**Study design/population studied:**

Critical reflections on a multi-disciplinary and multi-facet independent evaluation of a national program to implement electronic health record systems into 12 ‘early wave’ NHS hospitals in England.

**Findings:**

Our initial plan was to employ a mixed methods longitudinal ‘before-during-after’ study design. We however found this unsustainable in the light of fluxes in policy, contractual issues and over-optimistic schedules for EHR deployments. More importantly, this research design failed adequately to address the core of multi-faceted evolving EHRs as understood by key stakeholders and as worked out in their distinct work settings. Thus conventional outcomes-centric evaluations may not easily scale-up when evaluating transformational programs and may indeed prove misleading. New assumptions concerning the implementation process of EHR need to be developed that recognize the constantly changing milieu of policy, product, projects and professions that are inherent to such national implementations. The approaches we subsequently developed substitute the positivist view that EHR initiatives are self-evident and self-contained interventions, which are amenable to traditional quantitative evaluations, to one that focuses on how they are understood by various stakeholders and made to work in specific contexts. These assumptions recast the role of evaluation towards an approach that explores and interprets processes of socio-technical change that surround EHR implementation and adoption as seen by multiple stakeholders.

**Conclusions and policy implications:**

There is likely to be an increase in politically-driven national programs of reform of healthcare based on information and communication technologies. Programs on such a scale are inherently complex with extended temporalities and extensive and dynamic sets of stakeholders. They are, in short, different and pose new evaluation challenges that previously formulated evaluation methods for health information systems cannot easily address. This calls for methodological innovation amongst research teams and their supporting bodies. We argue that evaluation of such system-wide transformation programs are likely to demand both breadth and depth of experience within a multidisciplinary research team, constant questioning of what is and what can be evaluated and how, and a particular way of working that emphasizes continuous dialogue and reflexivity. Making this transition is essential to enable evaluations that can usefully inform policy-making. Health policy experts urgently need to reassess the evaluation strategies they employ as they come to address national policies for system-wide transformation based on new electronic health infrastructures.

